# Tyrosine Amino Acid as a Foulant for the Heterogeneous Anion Exchange Membrane

**DOI:** 10.3390/membranes13100844

**Published:** 2023-10-22

**Authors:** Anastasiia Kharina, Tatiana Eliseeva

**Affiliations:** Department of Analytical Chemistry, Voronezh State University, 1 Universitetskaya pl., Voronezh 394018, Russia; kharina@chem.vsu.ru

**Keywords:** tyrosine, organic fouling, anion exchange membrane, electrodialysis, transport, chemical and electrochemical cleaning

## Abstract

The features of organic fouling have been revealed for highly basic anion exchange membranes during prolonged electrodialysis in solutions containing the aromatic amino acid tyrosine. With increased operation time when using MA-41 heterogeneous membranes in tyrosine solution, an increase in hydrophobicity and roughness characteristics of the material surface is detected. A reduction in tyrosine flux through the membrane occurs which is caused by its pores plugging and deposition of the amino acid at the membrane surface induced by tyrosine adsorption and local supersaturation of the solution in the membrane phase. The long-term contact of the anion exchange membrane with a solution of tyrosine leads to some structural changes in the anion exchange material. An accumulation of the studied amino acid with phenolic fragment and tyrosine oxidation products (DOPA, DOPA-quinone) is found and confirmed by IR- and UV-spectroscopy techniques. The organic fouling is accompanied by an increase in density and a decrease in moisture content of the studied membrane. A comparative analysis of the chemical and electrochemical cleaning results for fouled samples of the MA-41 membrane demonstrates a partial restoration of the material transport characteristics using electrochemical cleaning in the intensive current mode of electrodialysis. The best efficiency of regeneration is reached when carrying out chemical cleaning with a solution of hydrochloric acid, providing almost complete restoration of the membrane characteristics.

## 1. Introduction

The great interest in amino acid chemistry, synthesis, and application is driven by their essential physiological functions and properties. The production of amino acids grows up rapidly and requires efficient procedures for their recovery from mixtures, purification, and concentration. The reagentless, environmentally and economically feasible method of electrodialysis with ion exchange membranes is applied to the purification of amino acids from inorganic impurities (such as mineral salts) [1,2,3,4,5,6,7], separation of amino acids and carbohydrates [5,8], separation of amino acid mixtures [9,10,11], conversion of amino acid hydrochlorides into amino acids [12,13,14], and concentration of amino acid solutions [15,16].

Some of these tasks can be solved not only using conventional electrodialysis, but also using electrodialysis with bipolar membranes, ion substitution electrodialysis, and other electromembrane methods. It should be noted that diffusion dialysis, Donnan dialysis, and neutralization dialysis with ion exchange membranes are also used for amino acid separation [17,18,19].

The features of amino acid transport and conditions for their separation from various solutions using electrodialysis and dialysis with ion exchange membranes have a significant influence on the efficiency of the processes. The interactions of the components in the system membrane–solvent–solute are rather complex in zwitterlyte-containing solutions. Along with the general patterns of amino acid mass transfer through ion exchange membranes under the action of an electric field gradient and/or a concentration gradient, some specific phenomena in membrane systems, which are determined primarily by the nature of interacting components, have an important influence on separation performance. Among such phenomena are those that lead to a decrease in the efficiency of electrodialysis and dialysis, in particular, the fouling of ion exchange membranes by treated solution components.

Under the influence of large organic ions, colloidal particles, and some inorganic compounds, as well as with the participation of microorganisms, fouling—a change in the intrinsic properties of ion exchange materials—can occur [20].

The fouling process can be reversible, partially reversible, and irreversible, depending on the degree of restoration of ion exchange material properties after regeneration (cleaning) [20].

According to the localization of fouling, two types can be distinguished:

- “Surface” fouling—the formation of a film at the surface of the membrane, caused by the deposition of sparingly soluble mineral or organic compounds [21].
- “Internal” fouling—the immobilization of multiply charged and/or large counter ions in the membrane phase [22].

Fouling almost always occurs during electrodialysis, but the effect of this phenomenon on the process is not the same; rather, it depends on the type of membranes used, on the nature and concentration of the foulants (substances present in the liquid medium), and on the experimental conditions.

The influence of various types of foulants in electromembrane processes is considered in [22,23,24]. Mechanisms of inorganic, organic, and biological fouling occurring in an electro membrane system are discussed currently in the literature [25,26,27,28,29]. Precipitation, deposition of colloidal particles, electrostatic and hydrophobic interactions, and formation of coordination compounds, as well as biological processes, are indicated among the predominant mechanisms of fouling for ion exchange membranes.

In particular, the phenomenon of organic fouling of ion exchange membranes, which leads to a decrease in the efficiency of electrodialysis separation of amino acids, is discussed in some studies [30,31,32,33]. A few works deal with a study of anion exchange membrane fouling by aromatic amino acids [34,35]. It is shown that fouling of heterogeneous anion exchange membranes by phenylalanine occurs at current densities close to the limiting one, and can be characterized by the increase in membrane voltage with time. The hydroxyl ions generated in the overlimiting regime enter into the anion exchange membrane phase and can “wash out” phenylalanine zwitterions by recharging them into anions [35]. It is stated that fouling in phenylalanine-containing solutions occurs due to electrostatic interactions (ion exchange) and also as a result of amino acid ion adsorption induced by hydrogen bonds between functional groups, and van der Waals forces. Phenylalanine zwitterions can also be bound to the membrane phase due to hydrophobic interactions of their benzene rings with benzene rings of amino acid anions being sorbed by the membrane. Hydrophobic interactions of the phenylalanine side chain with the polymer matrix of ion exchange resin are also a cause of fouling [34].

In this paper, we consider the alterations in the physicochemical, structural, and electrochemical characteristics of the anion exchange membranes during their long-term operation tests in the electrodialysis of tyrosine (4-hydroxyphenylalanine) solution. Tyrosine is an aromatic amino acid with phenolic fragment in the side chain. The novelty of this study consists in revealing the influence of such a side chain on anion exchange membrane fouling. The organic fouling of phenylalanine has been considered in our previous study [34]. The electrochemical regeneration makes it possible to fully restore membrane properties. Tyrosine is a low-soluble amino acid and it can also be oxidized at the surface and inside the membrane phase. We suppose the catalytic effect of anion exchange membrane functional groups in this process. The real interest of this study is the confirmation of tyrosine deposition at the surface and oxidation inside the membrane.

The propensity of anion exchange membranes to fouling with organic ampholytes, such as aromatic amino acids, requires not only the elucidation of the causes of this phenomenon and its mechanisms, but also the development of cleaning procedures. One of the tasks of this study is to compare some approaches to membrane regeneration such as chemical cleaning with various reagents and electrochemical regeneration in intensive current mode of electrodialysis.

## 2. Materials and Methods

### 2.1. Materials

The object of this study is tyrosine. The L-optical isomer of this aromatic amino acid was used in the experiments.

Tyrosine is a non-essential weak-acidic aromatic amino acid, a zwitterlyte. One of the most important features of zwitterlytes is the simultaneous presence in the solution of anions, cations, and zwitterions that mutually influence each other; the proportion of various forms depends on the pH of the medium. This significantly complicates the process of mass transfer through ion exchange membranes. Besides this, tyrosine has a phenolic group in the side chain that can lead to the special behavior of this amino acid during its contact with anion exchange materials.

Some characteristics of tyrosine are presented in Table 1.

In the electrodialysis cell, heterogeneous anion exchange membrane MA-41 (A) and cation exchange membrane MK-40 (C) were used. The matrix of C and A is made of a copolymer of styrene and divinylbenzene. The functional groups of the MA-41 membrane are quaternary amino groups. Sulfo groups are grafted onto the MK-40 membrane matrix.

Moreover, the heterogeneous bipolar MB-2 membrane (B) has been chosen, the anion exchange and cation exchange layers of which are identical to the anion exchange and cation exchange membranes used in this work, respectively. All used membranes were produced by OCC Shchekinoazot LLC, Pervomaisky, Russia. They are commercially available. The characteristics of the membranes are known from the manufacturer [37] and are presented in Table 2.

### 2.2. Methods

A seven-compartment laboratory electrodialysis cell with alternating cation exchange and anion exchange membranes is used. The fourth compartment of the electrodialysis cell is formed of bipolar and anion exchange membranes. The anion exchange layer of the bipolar membrane is turned to the anode, and the cation exchange layer to the cathode.

The height of the electrodialysis channel is 20 cm, and the width is 1 cm. The linear velocity of the solution is 0.1 cm s ^−1^.

**Figure 1:**
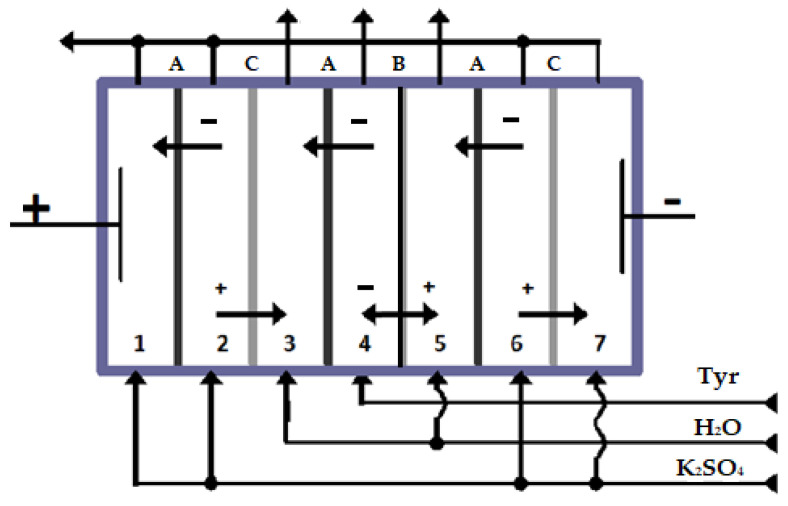
Scheme of the seven-compartment electrodialysis cell. ‘+’—cation, ‘−’—anion, E—electrodes.

Electrodialysis was realized at a constant current. The study was carried out after reaching a quasi-equilibrium state, which was established within 1 h and was detected when the voltage fluctuations had ceased. The electrodialysis cell (Figure 1) is equipped with a platinum anode and a high-alloy steel cathode. A model tyrosine solution (obtained from Sigma-Aldrich, Burlington, NJ, USA) at a concentration of 0.0025 M was supplied to the 4th compartment of the electrodialyzer. Compartments 3 and 5 were supplied with deionized water. The concentration of K_2_SO_4_ solution (obtained from Vekton-Center LLC, Voronezh, Russia) being fed to compartments 1, 2, 6, and 7 of the cell was 40 times higher than the concentration of the solution supplied to the 4th compartment. A quantitative analysis of solutions in compartments 3, 4, and 5 was carried out.

The pH value of the model amino acid solution being supplied into the fourth compartment was set close to its isoelectric point and varied in the range of 5.5–5.8. The pH of the solutions was measured using an I-160MI ionometer (RPA “Izmeritelnaya Tekhnika”, Moscow, Russia).

The concentration of tyrosine in the samples was measured using the spectrophotometry method according to the procedure [38].

Two different approaches to membrane regeneration were realized. Chemical cleaning of the ion exchange membranes was accomplished using NaOH (0.1 M), HCl (0.1 M), and NaOH (0.05 M) + NaCl (0.05 M) solutions (obtained from Vekton-Center LLC, Voronezh, Russia). Another approach to membrane regeneration was an electrochemical procedure of regeneration in the intensive current mode (current density and flow velocity in the 4th compartment 3–4 times higher than in the long-term experiment) using facilitated migration [5] with hydroxyl ions generated by the bipolar membrane during electrodialysis.

The calculation of tyrosine flux through the anion exchange membrane from compartment 4 into compartment 3 of the electrodialysis cell (Figure 1) is performed as follows:

(1) $$J=C\cdot V\cdot \tau^{-1}\cdot S^{-1},$$

where *J* is the flux of a component through an ion exchange membrane, mol·cm^−2^·s^−1^; *C* is the concentration of the solution, mol dm^−3^; *V* is the volume, dm^3^; *τ* is the sampling time, s; and *S* is the working area of the ion exchange membrane, cm^2^.

The effective transport numbers for hydroxyl ions were measured using an electro-analytical procedure during electrodialysis and calculated according to the following equation:

(2) $$T=z\cdot F\cdot J\cdot i^{-1},$$

where *T* is the effective transport number; *F* is the Faraday constant, C·mol^−1^; *z* is the charge number of ions; *i* is the current density, A·cm^−2^; and *J* is the flux of OH^−^ -ions, mol·cm^−2^·s^−1^.

Fouled membranes were obtained in a long-term operation test during electrodialysis in a model tyrosine solution (0.0025 M) within 60 h. The study of the fouling phenomenon was also carried out under static conditions by bringing a membrane sample into contact with the solution, within 50 days.

The alterations in the state of the anion exchange membrane surface that occur during its long-term operation test in electrodialysis of solutions containing tyrosine were analyzed using atomic force microscopy with a Solver P47 Pro scanning probe microscope (NT-MDT LLC, Moscow, Russia). The semi-contact method was used. The surface of the samples, which were dried at 50 °C, was scanned with an HA_FM NSG20 cantilever (NT-MDT LLC, Zelenograd, Russia). The cantilever oscillation frequency is 107.2 kHz. The experiments were carried out in air at a temperature of 25 ± 1 °C. Two-dimensional and three-dimensional digital images of the surface were obtained with a vertical resolution of at least 1 nm and a horizontal resolution of up to 40 nm. The processing of microphotographs is based on the analysis of standard arithmetic average surface parameters using the FemtoScanOnline program (femtoscan.2.4.25, 42.7 Mb) [39].

In this work, the surface topography for ion exchange membranes performed in a long-term operation test in tyrosine solutions was estimated using scanning electron microscopy with a JSM-6380 LV JEOL microscope (JEOL Ltd., Tokyo, Japan). In the process of pretreatment, ion exchange membranes were dried at 50 °C. To increase the contrast of micrographs, the surface of air-dry membranes in a vacuum of 10^−1^ mm Hg ion bombardment in a diode system was covered with gold using a Fin Coat 1100 installation at a constant voltage. At the same time, the thickness of the gold coating was varied from 1 × 10^−7^ to 2 × 10^−7^ m. A visual comparative analysis was carried out based on the obtained micrographs of the surface of samples in various ionic forms [40].

The density of wet pristine ion exchange membrane in the studied forms, as well as the density of fouled samples, was determined using the following express procedure. Two solvents are chosen; the density of the first one should be higher and of the second one lower than the density of the membrane. To a solvent with a lower density (carbon tetrachloride (Vekton-Center LLC, Voronezh, Russia)) containing the membrane under study, a solvent with a higher density (toluene (Vekton-Center LLC, Voronezh, Russia)) is added until the membrane sample begins to float. The density of the solvent mixture was measured to an accuracy of 10^−3^ using the areometer to determine the density of the membrane. The probability of these solvents’ uptake by the membrane was negligible in the conditions of the experiment [41].

The static contact angle of surface wetting for the MA-41 membrane after performance in tyrosine solutions was measured according to the procedure [42].

The moisture content of ion exchange membranes was determined by drying at a temperature of 50 °C to constant weight. The mass fraction of water in the membrane is calculated as the ratio of the difference between the mass of the wet membrane and the mass of the membrane after drying to the mass of the wet membrane.

To measure the electrical conductivity of the membranes in the studied forms using the contact–difference method, samples of two or three membranes being equal in area were placed in a two-electrode clamping cell made of organic glass included platinum electrodes with an area of 1 cm^2^, between which the membranes were fixed under the pressure of a weight of 1 kg. The measurements were carried out at a temperature of 25 °C. 

We used the distilled water in the cell to measure the conductivity of the anion exchange membrane in tyrosine form to exclude any influence of other ions. In the preliminary tests we found that the membrane conductivity values measured using the contact–difference method in individual tyrosine solutions are close to the values in distilled water. The use of the contact–difference method [43] makes it possible to calculate the resistance of the membrane without the resistance of the transition of the electrode–solution–membrane boundaries. In this case, the true value of the electrical conductivity of the membrane was determined from the difference in the active electrical resistances of three and two samples of the membrane [44,45]. The electrical conductivity was recorded over the wide AC frequency range to exclude effects that could affect the conductivity of the membrane at the definite value of AC frequency [43].

The electrical conductivity of ion exchange membranes is calculated taking into account the membrane thickness (*l*) measured with a micrometer using the formula:

(3) $$\chi_{m}=l\cdot S^{-1}\cdot {R_{a}}^{-1}$$

where *χ_m_* is the electrical conductivity of the membrane, Ohm^−1^·m^−1^, *l* is the membrane thickness, m; *S* is the area of the membrane, m^2^; and *R_a_* is the active resistance measured by the device, Ohm.

Structural changes in the ion exchange material applied in tyrosine-containing solutions for a long time were studied using the method of IR-spectroscopy. The IR spectra of the AV-17-8 anion exchanger in various forms (fresh and fouled by tyrosine) were obtained. AV-17-8 is the resin which is used for the MA-41 membrane production. Potassium bromide crushed to powder was applied to make tablets with the powdered anion exchanger. The tablets were pressed using special equipment at 25 °C under a pressure of 1.5 MPa. In the absorption mode, the tablets with potassium bromide were scanned at a temperature of 25 °C using a Vertex 70 device (BRUKER Corporation, Billerica, MA, USA) with a single beam scheme. The mass ratio of the background substance and a studied sample was 100:1 [46,47].

## 3. Results and Discussion

The stable functioning of membranes in electrodialyzers is one of the most important requirements for the application of membrane technologies in industry. The organic fouling of the surface and the volume of ion exchange membranes often hinders such functioning when working with solutions containing organic compounds. The anion exchange membranes are more susceptible to fouling by organics, in particular aromatic amino acids [34]. Therefore, changes in the electrochemical, transport, structural, and other characteristics of anion exchange membranes during their operation in solutions containing tyrosine (an aromatic amino acid with hydroxybenzene fragment) are considered.

### 3.1. Alterations in the Surface Structure of the Anion Exchange Membrane 

The alterations in the surface morphology of anion exchange membrane MA-41 in long-operation tests were evaluated using the microprofiles of the samples’ surface. Membranes in hydroxyl, Tyr («pristine» membrane) forms, and fouled membranes, after contact with a solution of Tyr (0.0025 M) for 50 days, as well as samples of membranes regenerated using different solutions, were compared.

**Figure 2:**
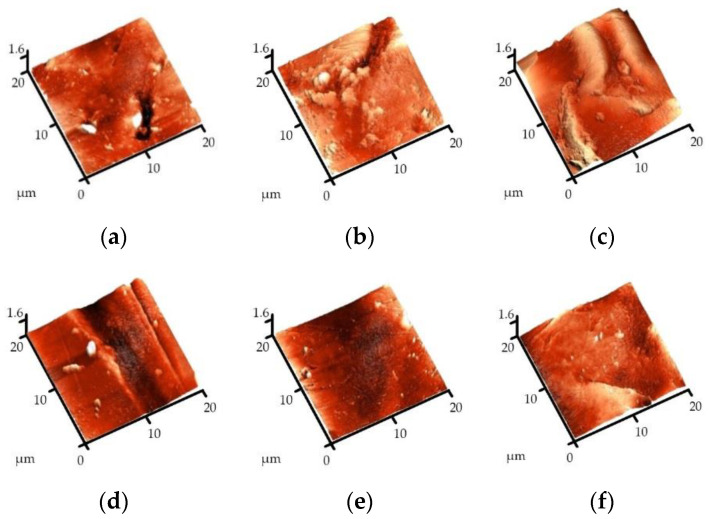
Three-dimensional images of the surface of the MA-41 heterogeneous membrane: (**a**) OH^−^-form, (**b**) Tyr-form, and (**c**) fouled membrane after 50 days of contact with a fresh Tyr solution; membrane regenerated using (**d**) HCl, (**e**) NaOH, and (**f**) NaOH + NaCl solutions (Scanned field—(20 × 20) × 1.6 μm).

The most significant alterations in the surface morphology of the studied ion exchange membranes are evident when comparing microphotographs of the surface of the hydroxyl form of MA-41 with the amino acid form and with the fouled sample of the membrane, which has been in contact with a solution containing tyrosine (Figure 2a–c).

It was found that after contact with the aromatic amino acid solution, an increase in the roughness of the membrane surface is observed, which is due to the sorption of large tyrosine ions on the surface.

A long-term operation of the anion exchange membrane under consideration in the aromatic amino acid solution leads to an additional increase in the membrane surface roughness and plugging of the membrane pores.

The regeneration (cleaning) of membranes with a solution of hydrochloric acid (Figure 2d) ensures the restoration of parameters characterizing the surface roughness. A sodium hydroxide solution (Figure 2e) and a mixed solution of sodium hydroxide and sodium chloride (Figure 2f) show less efficiency in regeneration, and the surface roughness after cleaning is close to the characteristics of a «pristine» membrane in amino acid form. Table 3 demonstrates the roughness parameters of the anion exchange membrane MA-41 in different forms.

**Figure 3:**
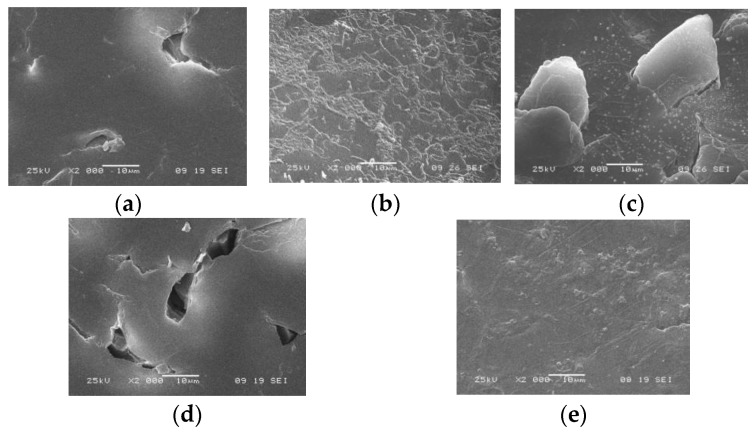
Microphotographs of the surface of the MA-41 membrane in various forms: (**a**) OH^−^, (**b**) Tyr, (**c**) fouled membrane after 50 days of contact with a fresh Tyr solution; membrane regenerated using (**d**) a HCl solution and (**e**) a NaOH solution (magnification ×2000).

The microphotographs of the MA-41 membrane surface obtained using the method of scanning electron microscopy also show changes in the surface after the sorption of tyrosine and after prolonged contact of the membrane with a solution of this aromatic amino acid. The regeneration solutions that were most effective in preliminary tests (HCl and NaOH) were compared. Figure 3 presents microphotographs of the surface of the MA-41 anion exchange membrane in various forms obtained using scanning electron microscopy.

As can be seen from the presented microphotographs, for the samples of the studied anion exchange membranes after long-term operation tests in tyrosine solution, an increase in the geometric inhomogeneity of the membrane surface, as well as plugging of its pores, are observed.

The organic fouling phenomenon for the anion exchange membrane MA-41 in an aromatic amino acid solution has been revealed in [34] for phenylalanine (a benzyl-containing side chain) and explained by ion–ion interactions and hydrophobic interactions of the benzene rings of the amino acid and the styrene–divinylbenzene matrix. Such interactions are also possible for other aromatic amino acids.

However, in the considered case of tyrosine (4-hydroxyphenylalanine), the low solubility of the studied amino acid should be noted. It complicates the phenomenon of fouling by the deposition of a sparingly soluble tyrosine at the surface and inside the ion exchange membrane due to a local supersaturation of the solution caused by the uptake of this amino acid [48,49].

The analysis of microphotographs shows the possibility of regeneration of the surface of the MA-41 membrane fouled by tyrosine. After treatment of the membranes with a NaOH solution, the surface is not completely free from tyrosine. The efficiency of the HCl cleaning solution is greater.

The study of changes in the structure of the surface of anion exchange membranes in various forms using scanning electron and atomic force microscopy makes it possible to visually fix the fouling of ion exchange membranes operating in solutions containing tyrosine. The application of a hydrochloric acid solution is the most effective approach for membrane surface regeneration. This is explained by the fact that the solubility of tyrosine increases significantly at low pH [50].

### 3.2. Alterations in the Electrical Conductivity of the Anion Exchange Membrane

**Figure 4:**
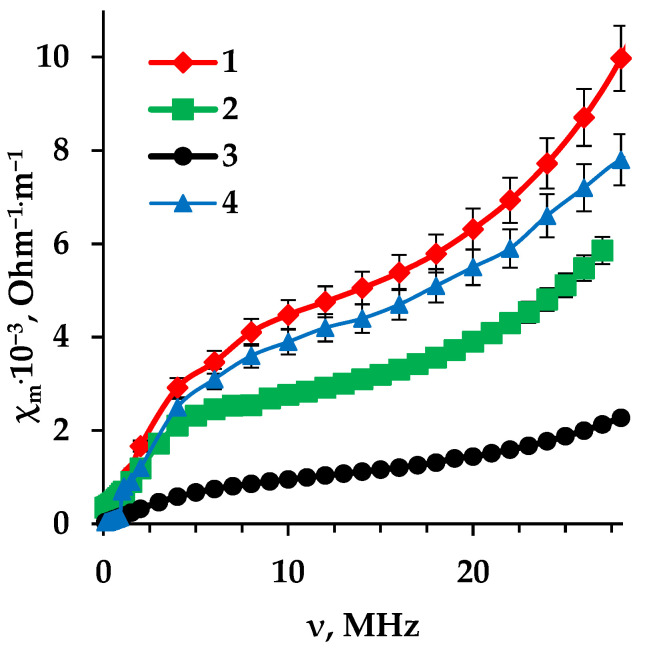
Dependence of conductivity of the MA-41 membrane on AC frequency: 1—OH-form, 2—Tyr-form («pristine» membrane), 3—fouled membrane after 50 days of operation in Tyr solution, 4—membrane regenerated using a HCl cleaning solution.

The electrical conductivity of ion exchange membranes determines their suitability for application in electromembrane processes. To reveal the changes in the characteristics of anion exchange membranes operating in the aromatic amino acid solution, we measured the specific electrical conductivity of the MA-41 membrane in various forms: OH, Tyr-form («pristine» membrane), fouled membrane after prolonged contact with the amino acid solution during 50 days, as well as membrane regenerated using a HCl (0.1 M) solution. Figure 4 shows the dependences of the electrical conductivity of the MA-41 membrane on the frequency of alternating current (AC).

The dependence of the specific electrical conductivity of the MA-41 membrane in various forms on the frequency of alternating current consists of three regions. The effect of alternating current frequency on electrical conductivity is explained by the Debye–Falkenhagen effect [51]. An increase in the electrical conductivity of the membrane with an alternating current frequency in the initial region is caused by a decrease in the influence of the relaxation effect while maintaining the effect of cataphoretic braking. With a further increase in the frequency of the alternating current, only the cataphoretic effect is observed (region 2). Upon reaching higher values of the AC frequency above 15 MHz, an increase in membrane conductivity is revealed, since in ion exchange membranes at a given frequency, the movement of ions is not hindered by the effect of cataphoretic braking, and ions with the same charge sign move in the direction of the electric field due to convective electrical conductivity in the pore solution of the ion exchange membrane. The obtained dependences of electrical conductivity on alternating current frequency for pristine and fouled membranes are similar within the measurement error up to an alternating current frequency of 1.25 MHz when the influence of cataphoretic and relaxation effects is significant. However, one can find a difference at a higher frequency. So there is fouling influence on the membrane resistance.

The electrical conductivity of the anion exchange membrane in the hydroxyl form is maximum in the considered series (Figure 4). After sorption of the amino acid, for the «pristine» membrane the electrical conductivity is lower than for the membrane in the hydroxyl form. The decrease in the electrical conductivity of the anion exchange membrane is associated with a significantly lower mobility of amino acid ions compared to the mobility of hydroxyl ions.

The minimum conductivity was recorded for a highly basic anion exchange membrane after its long-term contact (50 days) with a solution of the aromatic amino acid under study. This is due to the organic fouling of the membranes, which leads to the effect of functional groups shielding, and the plugging of the pores of ion exchange membranes by the aromatic amino acid. Therefore, it is necessary to use a procedure to reduce membrane fouling in order to increase their service life during the electrodialysis of solutions containing tyrosine. The chemical regeneration of fouled samples using an acid solution provides almost complete restoration of the electrical conductivity value for the membrane.

**Figure 5:**
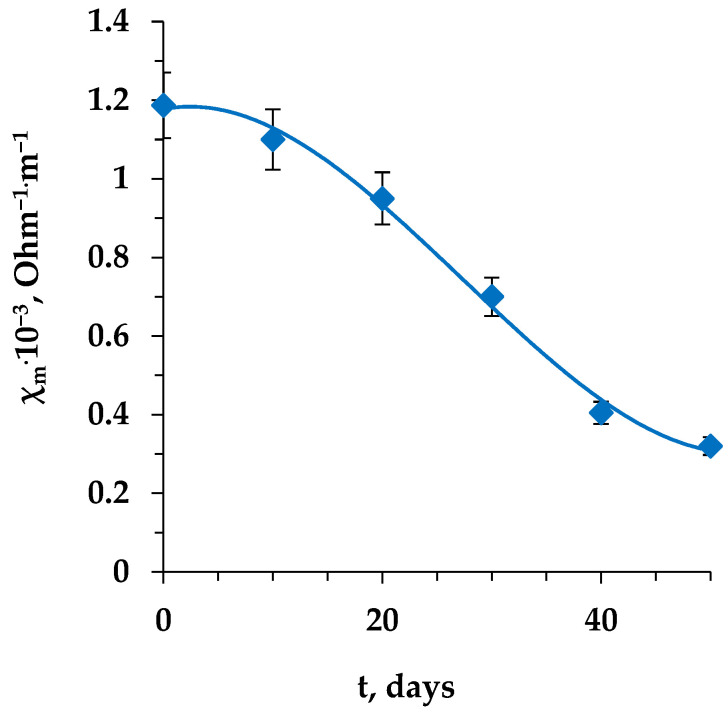
The dependence of the electrical conductivity of the MA-41 membrane on the operating time in the electrodialysis of tyrosine solution.

In this work, the kinetic curve for the electrical conductivity of the membrane at the frequency of alternating current 2 MHz during long-term contact with a tyrosine solution was additionally studied (Figure 5).

The electrical conductivity of the MA-41 membrane decreases with increasing operating time during the electrodialysis of tyrosine solution without application of intermittent regeneration (cleaning). A more significant decrease in electrical conductivity is observed after 10 days of contact with a solution of the amino acid under study.

### 3.3. Alterations in Some Physicochemical Characteristics of the Anion Exchange Membrane

For the samples of the studied MA-41 anion exchange membrane, we determined the values of contact angle of wetting, density, and moisture content. These characteristics are considered for membranes in hydroxyl form, amino acid form («pristine» membrane), fouled membrane after prolonged contact with tyrosine solution for 50 days, as well as membranes regenerated using HCl (0.1 M) and NaOH (0.1 M) solutions (Table 4).

The density of the anion exchange membrane in Tyr-form and the contact angle of wetting of its surface are greater than in the hydroxyl form. The contact of the membrane with the amino acid solution for 50 days leads to an additional increase in density and contact angle of wetting.

A comparative analysis of the moisture content of membrane samples in hydroxyl and amino acid forms indicates a decrease in the moisture content of the membrane after sorption of the amino acid. Monitoring the moisture content of the MA-41 ion exchange membrane during contact with an individual Tyr amino acid solution for 50 days shows the absence of a significant change in moisture content with an increase in operation time.

The observed changes in moisture content, density, and contact angle occur as a result of the accumulation in the membrane phase of large ions of tyrosine, causing a decrease in the hydrophilicity of the surface and moisture content of the membrane.

For membrane samples regenerated using a hydrochloric acid solution, the density, contact angle, and moisture content are close to the values of the membrane in the hydroxyl form. Regeneration using an alkali solution is less effective. The studied physicochemical characteristics of the membrane regenerated using this solution are close to the values for the sample in the amino acid form.

### 3.4. Changes in the Transport Characteristics of the Anion Exchange Membrane

**Figure 6:**
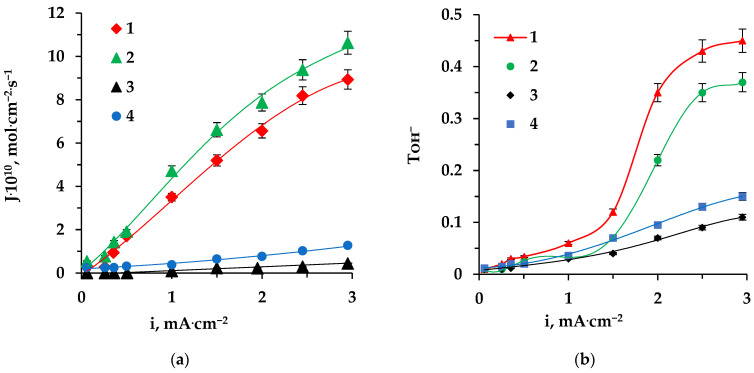
Dependences of Tyr fluxes through the MA-41 membrane (**a**) and OH^−^-ions effective transport number for this membrane (**b**) on the current density in the electrodialysis of a Tyr solution: 1—membrane after reaching a steady state in electrodialysis (pristine membrane), 2—membrane after 50 h of operation, 3—membrane after 60 h of operation, 4—regenerated membrane.

The dependence of amino acid fluxes through ion exchange membranes in an electromembrane system with alternating cation and anion exchange monopolar membranes on current density is non-linear [5]. The dependence of amino acid flux through the anion exchange membrane on current density recorded in the system with a bipolar membrane (Figure 1) revealed no decrease in amino acid flux during the electrodialysis of an individual tyrosine solution (Figure 6a).

This is due to the generation of H^+^ and OH^−^ ions at the interphase boundary of a bipolar membrane. In particular, the protolithic reaction of Tyr zwitterions with OH^−^ ions leads the appearance of amino acid anions in compartment 4 of the apparatus even at low currents. Tyrosine anions migrate through the anion exchange membrane. A comparative analysis of the dependences in Figure 6a shows that Tyr flux through the membrane that has been used for 50 h without its regeneration reaches an even higher value compared to a pristine sample. Under the influence of the mass transfer of large-sized organic ions, porosity changes, so pores of a higher radius appear in comparison with a pristine membrane [52]. An increase in the pore size of the anion exchange material matrix during electrodialysis in contact with a tyrosine solution leads to an increase in the diffusion permeability of the membrane, the volume fraction of the inter-gel phase, and the geometric heterogeneity of the membrane surface. Similar changes in structure of the anion exchange material are described in the literature, which provides data on alterations that occur during prolonged contact with a solution containing sodium dihydrogen phosphate [53]. On the other hand, the detected increase in amino acid flux may be associated with an increase in contribution of electroconvection to the mass transfer of components through the membrane as a result of an increase in the roughness and the hydrophobicity of the membrane surface [2,32], confirmed by an increase in the wetting contact angle (Table 4).

However, with the subsequent use of the membrane in electrodialysis, the flux of tyrosine through MA-41 decreases (Figure 6a). The decrease in amino acid mass transfer may be due to the shielding of membrane functional groups by amino acid ions and the plugging of membrane pores, complicating the reaction of water dissociation due to a decrease in the catalytic activity of the membrane functional groups. Moreover, amino acid deposition in the membrane phase occurs that is caused by the low solubility of tyrosine and the local supersaturation of the internal solution.

This fact is indirectly confirmed by the decrease in the effective transport number of OH-ions during the electrodialysis of the Tyr solution (Figure 6b).

With prolonged electrodialysis without membrane regeneration, an additional decrease in the values of the effective transport number for OH-ions is revealed. It confirms the organic fouling of membranes.

In order to develop useful approaches for the cleaning of fouled anion exchange membranes, the efficiency of electrochemical regeneration in the intensive current mode of electrodialysis (current density and flow velocity in the 4th compartment 3–4 times higher than in the long-term experiment) is considered. In the process of zwitterlyte solution electrodialysis, it is possible to regenerate membranes “in situ” not applying additional reagents, since the amino acid in an intensive current mode can be “washed out” from the anion exchange membrane phase using conjugated flux with hydroxyl ions formed during the dissociation of water occurring at the interphase boundary membrane–solution. We have found that such electrochemical regeneration in electrodialysis of phenylalanine solution is very effective [34] and fully reduces the results of fouling. The special behavior of tyrosine leads to a significant decrease in electrochemical regeneration efficiency. However, we have found that acidic cleaning is very effective (Figure 6). This fact may be associated with the higher solubility of tyrosine in an acidic medium [50].

### 3.5. Changes in the Structural Characteristics of the Anion Exchange Material

**Figure 7:**
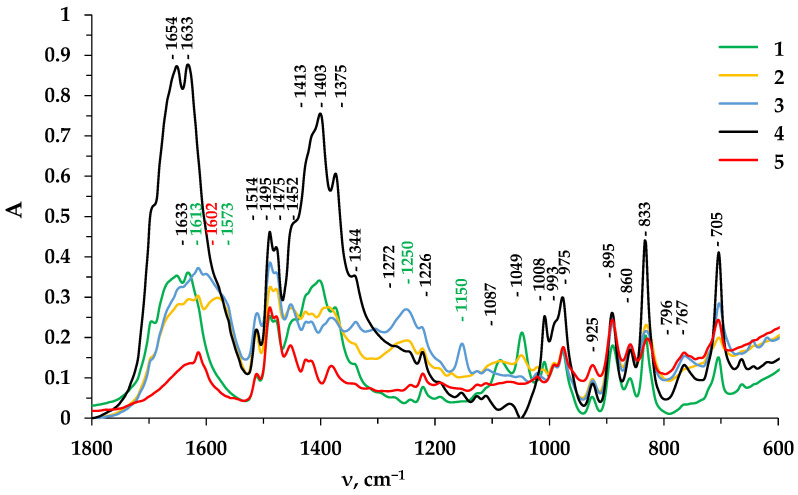
IR spectra of anion exchanger AV-17-8: 1—OH-form, 2—Tyr-form, 3—after prolonged contact with a Tyr solution during 50 days, 4—regenerated using a NaOH solution, 5—regenerated using a HCl solution.

Structural changes are considered after long-term operation tests of the anion exchange material AV-17-8 used in the manufacturing of the studied heterogeneous anion exchange membrane MA-41, in the structure of which there is an inert filler (polyethylene), which complicates the structural group analysis. We also compared the efficiency of the most promising among the considered regeneration solutions, namely, NaOH and HCl with a concentration of 0.1 M. Figure 7 shows the IR spectra of the anion exchanger in various ionic forms.

Absorption bands at 1150 cm^−1^ (phenolate ion of Tyr), and 1573 and 1250 cm^−1^ (carboxylate ion of Tyr) confirm the presence of an amino acid after its sorption in the phase of anion exchanger. In the IR spectrum of the anion exchanger AV-17-8, which has been in contact with a solution of an aromatic amino acid for a long time, in addition to absorption bands indicating the accumulation of an amino acid in the phase of the material, a new peak at 1602 cm^−1^ is detected. This absorption band corresponds to the stretching vibrations of the enol group, and it is indirect evidence of the appearance in the phase of the anion exchanger of a Tyr oxidation product, dioxyphenylalanine (DOPA). In this case, the anion exchanger, after long-term contact with the Tyr solution, acquires a dark brown color.

With prolonged contact of the anion exchange material with the aromatic amino acid solution, in addition to the interaction of the functional groups of the material with this type of organic ampholytes, as well as products of its chemical reaction, hydrophobic interactions between the aromatic rings of the amino acids and the polymer matrix of the anion exchange material are possible—π-π interactions with the formation of charge–transfer complexes.

A complete regeneration of the AV-17-8 anion exchanger operated in tyrosine solutions is not reached with a sodium hydroxide solution. The most effective regenerating solution is 0.1 M HCl. The absorption bands that reveal the presence of tyrosine and its oxidation products in the structure of the anion exchanger disappear. So, this regenerating solution is the optimal one among those considered. In addition, the use of regenerating solutions with an alkaline pH can cause a deamination reaction.

To reveal the occurrence of undesirable interactions in the aromatic amino acid–water–membrane system, solutions of desorption products from the anion exchange material in contact with an amino acid solution were studied using the method of spectrophotometry.

**Figure 8:**
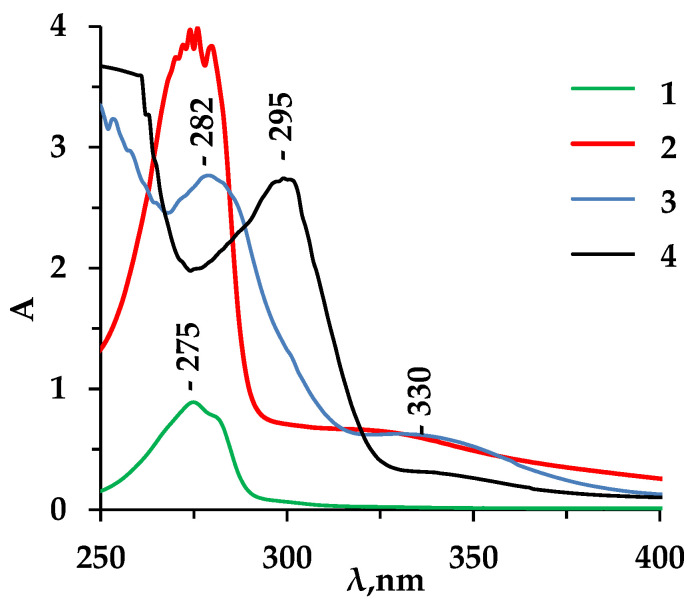
UV spectra of solutions: 1—Tyr (0.0005 M), pH = 6.01, 2—Tyr (0.0025 M), which was in contact with atmospheric oxygen for a long time, 3—HCl solution after the regeneration of the MA-41 membrane fouled by Tyr, 4—NaOH solution after the regeneration of the MA-41 membrane fouled by Tyr.

A comparative analysis of the UV spectra of Tyr solutions (0.0025 M), which were in contact with atmospheric oxygen for a long time at room temperature, as well as solutions obtained after the regeneration of an anion exchanger that had been in a solution of an aromatic amino acid for a long time, was carried out (Figure 8). In this case, solutions of hydrochloric acid (0.1 M) and sodium hydroxide (0.1 M) were used as regeneration solutions. Figure 8 also shows the UV spectra of the freshly prepared solutions of the studied amino acid. The regenerating solutions after their use, as well as the solutions of the amino acid during long-term storage, acquired a dark brown color.

It was found that an additional absorbance band appears for the UV spectrum at λ = 330 nm. In the UV absorption spectrum of tyrosine solution stored at room temperature for two months, this maximum also appears which does not correspond to the amino acid.

**Figure 9:**
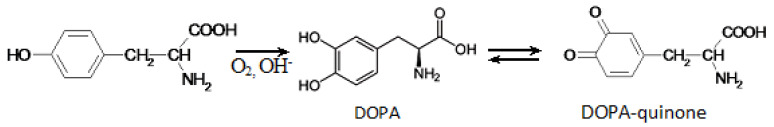
Oxidation of tyrosine to DOPA-quinone.

To explain the appearance of the additional absorption band in the obtained UV and IR spectra, the possible oxidation of tyrosine due to electron-donor substituents of the aromatic ring of the amino acid, which activate it, is considered. In oxidation, dihydroxyphenylalanine and its tautomeric ketone form, DOPA-quinone, can be formed in accordance with the scheme shown in Figure 9 [54,55].

According to the literature data [56], the UV absorption spectrum of melanin solution also shows an absorption maximum at λ = 330 nm, which may be indirect evidence of the formation of quinoid structures.

The oxidation of tyrosine is a process that proceeds slowly in time in solution. However, in a strongly alkaline medium in the phase of anion exchange material containing groups of quaternary ammonium bases, oxidation products of this amino acid are detected after a shorter period of membrane operation.

## 4. Conclusions

The features of organic fouling of a highly basic anion exchange membrane during electrodialysis operation tests in solutions containing the aromatic amino acid tyrosine have been estimated. Changes in the electrochemical, transport, structural, and other characteristics of the studied anion exchange materials have been found.

With prolonged operation tests of the MA-41 anion exchange membrane in electrodialysis, an increase in the hydrophobicity of the material surface was confirmed by an increase in the contact angle of wetting. An increase in the roughness of the membrane surface was also revealed. Moreover, with the fouling of the membrane, an increase in density and a decrease in the moisture content of the studied membrane were shown.

The detected increase in Tyr flux through the MA-41 during electrodialysis without intermittent membrane regeneration for 50 h compared to the pristine sample may be due to several reasons. An expansion of the pores of the anion exchange membrane in the electrodialysis of the tyrosine solution can lead to an increase in the diffusion permeability of the membrane, the volume fraction of the inter-gel phase, and the geometric heterogeneity of the membrane surface. On the other hand, the detected initial growth in amino acid flux with time may be associated with an increase in electroconvection impact in the mass transfer through the membrane as a result of an increase in the roughness and hydrophobicity of the membrane surface.

The following sharp reduction in tyrosine flux through the anion exchange membrane with time (more than 60 h) occurs because of tyrosine interactions with the membrane by various mechanisms, the plugging of the membrane pores, and the amino acid deposition in the membrane phase by reason of the local supersaturation of the internal solution caused by a low soluble compound.

A long-term contact of the anion exchange membrane with a solution of tyrosine leads to a change in the structure of the anion exchange material. An accumulation of amino acid tyrosine occurs in the membrane phase because of the ion exchange, ion adsorption due to intermolecular interactions induced by hydrogen bonds between functional groups, and van der Waals forces. Tyrosine zwitterions can also be involved in interactions of their benzene rings with the benzene rings of amino acid anions sorbed by the membrane. The interaction of the tyrosine benzene ring with the benzene rings of the anion exchange membrane matrix is also a reason of fouling. Moreover, an accumulation of tyrosine oxidation products (DOPA and DOPA-quinone) has been detected.

A comparative analysis of the chemical and electrochemical regeneration of the fouled samples of the MA-41 membrane shows a partial restoration of the transport characteristics of the membrane during electrochemical regeneration in the intensive current mode of electrodialysis. After chemical regeneration with an alkali solution, a partial restoration of the studied characteristics of the membrane is also observed. The highest efficiency is achieved when carrying out chemical regeneration with a solution of hydrochloric acid, in which almost complete restoration of the studied membrane characteristics occurs. This feature of membrane organic fouling reduction in tyrosine solution deals with a significant increase in this amino acid’s solubility in the HCl solution.

## Figures and Tables

**Table 1:** The structure and properties of tyrosine [36].

Amino Acid	Structure	pI	pK			Relative Molecular Weight	Solubility, g/100 mL H_2_O, 25 °C
			pK_1_	pK_2_	pK_R_		
Tyrosine (Tyr)	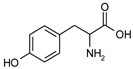	5.63	2.20	10.07	9.40	181.19	0.453

**Table 2:** Physical-chemical characteristics of the ion exchange membranes used [37].

Characteristic/Membrane	MK-40	MA-41	MB-2
Composite repeating unit	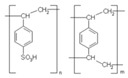	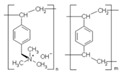	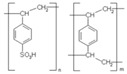 , 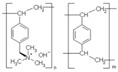
Tensile strength, MPa, ≤	11.9	11.0	5.5
Surface electrical resistance, Ohm·cm^2^, ≤	10.0	11.0	18.0

**Table 3:** Roughness parameters of the anion exchange membrane MA-41 in different forms: OH^−^ (1), Tyr-form (2), fouled membrane after 50 days of contact with a Tyr solution (3); membrane regenerated using HCl (4), NaOH (5), and NaOH + NaCl (6) solutions.

Roughness Parameters	Membrane					
	1	2	3	4	5	6
Peak-to-peak, nm	1277	1555	2009	1306	1435	1633
Root mean square roughness, nm	111	156	288	110	122	156

**Table 4:** Physicochemical characteristics of the MA-41 membrane in different forms.

Membrane Form	Contact Angle, θ, °	Density, ρ, g cm^−3^	Moisture Content, W, %
OH^−^	66.0 ± 0.9	1.053 ± 0.001	38.9
Tyr («pristine» membrane)	73.3 ± 1.6	1.064 ± 0.005	31.5
fouled membrane after 50 days of contact with Tyr solution	81.1 ± 0.8	1.071 ± 0.001	33.1
regenerated membrane (using HCl)	69.2 ± 0.7	1.055 ± 0.003	37.1
regenerated membrane (using NaOH)	74.2 ± 0.7	1.066 ± 0.002	32.2

## Data Availability

Not applicable.

## References

[1] Wang M., Kuang S., Wang X., Kang D., Mao D., Qian G., Cai X., Tan M., Liu F., Zhang Y. (2021). Transport of Amino Acids in Soy Sauce Desalination Process by Electrodialysis. Membranes.

[2] Eliseeva T., Kharina A. (2022). Desalination of Neutral Amino Acid Solutions in an Electromembrane System. Membranes.

[3] Wu J., Xu C., Zhang C., Wang G., Yan Y., Wu C., Wu Y. (2017). Desalination of L-Threonine (THR) Fermentation Broth by Electrodialysis. DWT.

[4] Aghajanyan A.E., Hambardzumyan A.A., Vardanyan A.A., Saghiyan A.S. (2008). Desalting of Neutral Amino Acids Fermentative Solutions by Electrodialysis with Ion-Exchange Membranes. Desalination.

[5] Elisseeva T.V., Shaposhnik V.A., Luschik I.G. (2002). Demineralization and Separation of Amino Acids by Electrodialysis with Ion-Exchange Membranes. Desalination.

[6] Choi J.-H., Oh S.-J., Moon S.-H. (2002). Structural Effects of Ion-Exchange Membrane on the Separation of L-Phenylalanine (L-Phe) from Fermentation Broth Using Electrodialysis. J. Chem. Technol. Biotechnol..

[7] Nikonenko V.V., Pismenskaya N.D., Pourcelly G., Larchet C., Yaroslavtsev A.B. (2013). Simulation of Transport Phenomena in the Systems with Ion-Exchange Membranes. Membranes and Membrane Technologies.

[8] Kharina A.Y., Charushina O.E., Eliseeva T.V. (2023). Organic Fouling of Anion-Exchange and Bipolar Membranes in the Separation of Amino Acids and Sucrose by Electrodialysis. Condens. Matter Interfaces.

[9] Shaposhnik V.A., Selemenev V.F., Polyanskaya N.N. (1990). Separation of Valine, Lysine and Glutamic Acid by Electrodialysis with Ion Exchange Membranes. J. Appl. Chem..

[10] Yuan F., Wang Q., Yang P., Cong W. (2016). Transport Properties of Amino Acid Ions at Isoelectric Point in Electrodialysis. Sep. Purif. Technol..

[11] Wang G., Zhang C., Sun M., Zhang X., Wu C., Wu Y. (2017). Separation of Mixed Amino Acids by BMED Process Using Porous SPES and SPSf Cation Exchange Membranes. Sep. Purif. Technol..

[12] Zhang Y., Chen Y., Yue M., Wang L. (2012). Production of l-Lysine from l-Lysine Monohydrochloride by Bipolar Membrane Electrodialysis. Desalination Water Treat..

[13] He J., Liu W., Hao J., Ma X., Zheng Z., Fang Y., Liang Y., Tian Z., Sun L., Li C. (2023). Bipolar Membrane Electrodialysis for Direct Conversion of L-Ornithine Monohydrochloride to L-Ornithine. IJMS.

[14] Bobreshova O.V., Kulintsov P.I., Bobrinskaya G.V., Balavadze E.M. (2001). Electrodialysis Conversion of L-Lysine Monochlorohydrate to L-Lysine. Sorptsionnye I Khromatographicheskie Protsessi.

[15] Eliseeva T., Krisilova E., Oros G., Selemenev V., Jacobs N.L. (2011). Arginine: Physico-Chemical Properties, Interactions with Ion-Exchange Membranes. Recovery and Concentration by Electrodialysis. Arginine Amino Acid.

[16] Buchbender F., Wiese M. (2018). Efficient Concentration of an Amino Acid Using Reactive Extraction Coupled with Bipolar Electrodialysis. Chem. Eng. Technol..

[17] Vasil’eva V.I., Goleva E.A. (2013). Selective Separation of Sodium Ions from a Mixture with Phenylalanine by Donnan Dialysis with a Profiled Sulfogroup Cation Exchange Membrane. Russ. J. Phys. Chem..

[18] Vasil’eva V., Goleva E., Pismenskaya N., Kozmai A., Nikonenko V. (2019). Effect of Surface Profiling of a Cation-Exchange Membrane on the Phenylalanine and NaCl Separation Performances in Diffusion Dialysis. Sep. Purif. Technol..

[19] Kozmai A., Porozhnyy M., Gil V., Dammak L. (2023). Phenylalanine Losses in Neutralization Dialysis: Modeling and Experiment. Membranes.

[20] Strathmann H. (2004). Ion-Exchange Membrane Separation Processes.

[21] Grossman G., Sonin A.A. (1973). Membrane Fouling in Electrodialysis: A Model and Experiments. Desalination.

[22] Korngold E., de Körösy F., Rahav R., Taboch M.F. (1970). Fouling of Anionselective Membranes in Electrodialysis. Desalination.

[23] Spettmann D., Eppmann S., Flemming H.-C., Wingender J. (2007). Simultaneous Visualisation of Biofouling, Organic and Inorganic Particle Fouling on Separation Membranes. Water Sci. Technol..

[24] Lindstrand V., Sundström G., Jönsson A.-S. (2000). Fouling of Electrodialysis Membranes by Organic Substances. Desalination.

[25] Dammak L., Fouilloux J., Bdiri M., Larchet C., Renard E., Baklouti L., Sarapulova V., Kozmai A., Pismenskaya N. (2021). A Review on Ion-Exchange Membrane Fouling during the Electrodialysis Process in the Food Industry, Part 1: Types, Effects, Characterization Methods, Fouling Mechanisms and Interactions. Membranes.

[26] Hansima M.A.C.K., Makehelwala M., Jinadasa K.B.S.N., Wei Y., Nanayakkara K.G.N., Herath A.C., Weerasooriya R. (2021). Fouling of Ion Exchange Membranes Used in the Electrodialysis Reversal Advanced Water Treatment: A Review. Chemosphere.

[27] Mikhaylin S., Bazinet L. (2016). Fouling on Ion-Exchange Membranes: Classification, Characterization and Strategies of Prevention and Control. Adv. Colloid Interface Sci..

[28] Apel P.Y., Velizarov S., Volkov A.V., Eliseeva T.V., Nikonenko V.V., Parshina A.V., Pismenskaya N.D., Popov K.I., Yaroslavtsev A.B. (2022). Fouling and Membrane Degradation in Electromembrane and Baromembrane Processes. Membr. Membr. Technol..

[29] Lee H.-J., Hong M.-K., Han S.-D., Cho S.-H., Moon S.-H. (2009). Fouling of an Anion Exchange Membrane in the Electrodialysis Desalination Process in the Presence of Organic Foulants. Desalination.

[30] Zheng Y., Jin Y., Zhang N., Wang D., Yang Y., Zhang M., Wang G., Lee S., Qu W. (2022). Recovery of N, N-Dimethylglycine (DMG) from Dimethylglycine Hydrochloride by Bipolar Membrane Electrodialysis. Chem. Eng. Process. Process Intensif..

[31] Suwal S., Doyen A., Bazinet L. (2015). Characterization of Protein, Peptide and Amino Acid Fouling on Ion-Exchange and Filtration Membranes: Review of Current and Recently Developed Methods. J. Membr. Sci..

[32] Eliseeva T., Kharina A. (2023). Current-Voltage and Transport Characteristics of Heterogeneous Ion-Exchange Membranes in Electrodialysis of Solutions Containing a Heterocyclic Amino Acid and a Strong Electrolyte. Membranes.

[33] Kattan Readi O.M., Gironès M., Nijmeijer K. (2013). Separation of Complex Mixtures of Amino Acids for Biorefinery Applications Using Electrodialysis. J. Membr. Sci..

[34] Bukhovets A., Eliseeva T., Oren Y. (2010). Fouling of Anion-Exchange Membranes in Electrodialysis of Aromatic Amino Acid Solution. J. Membr. Sci..

[35] Bukhovets A., Eliseeva T., Dalthrope N., Oren Y. (2011). The Influence of Current Density on the Electrochemical Properties of Anion-Exchange Membranes in Electrodialysis of Phenylalanine Solution. Electrochim. Acta.

[36] Jakubke H.-D., Jeschkeit H., Jakubke H.-D. (1977). Amino Acids, Peptides and Proteins: An Introduction.

[37] http://www.azotom.ru/ionoobmennye_membrany/.

[38] Silverstein R.M., Bassler G.C., Morrill T.C. (1974). Spectrometric Identification of Organic Compounds.

[39] Arutyunov P.A., Tolstikhina A.L., Demidov V.N. (1998). System of Parameters for the Analysis of Roughness and Microrelief of the Surface of Materials in Scanning Probe Microscopy. Ind. Lab. Diagn. Mater..

[40] DeStefanis A., Tomlinson A.A.G. (2001). Scanning Probe Microscopies: From Surface Structure to Nano-Scale Engineering.

[41] Bogatyrev V.L. (1968). Ion Exchangers in the Mixed Layer.

[42] Starov V.M., Zhdanov S.A., Kosvintsev S.R., Sobolev V.D., Velarde M.G. (2003). Spreading of Liquid Drops over Porous Substrates. Adv. Colloid Interface Sci..

[43] Badessa T.S., Shaposhnik V.A. (2017). Electrical conductance studies on ion exchange membrane using contact-difference method. Electrochim. Acta.

[44] Shaposhnik V.A. (1989). Kinetika Ėlektrodializa.

[45] Berezina N.P. (2009). Electrochemistry of Membrane Systems.

[46] Uglianskaia V.A., Chikin G.A., Selemenev V.F., Zavialova T.A. (1989). Infrakrasnaia Spektroskopiia Ionoobmennykh Materialov.

[47] Bellamy L.J. (1975). The Infra-Red Spectra of Complex Molecules.

[48] Muravyov D.N. (1979). Ion-Exchange Isothermal Supersaturation of Amino Acid Solutions. J. Phys. Chem..

[49] Muravyov D.N., Fesenko S.A. (1982). Ion Exchange in Supersaturated Solutions. Calculation of the Work of Formation of Critical Nuclei of Some Amino Acids from Supersaturated Solutions. J. Phys. Chem..

[50] Prokhorov A.M., Knuniants I.L. (1988). Khimicheskaia Ėntssklopediia: V Piati Tomakh.

[51] Zabolotskiĭ V.I., Nikonenko V.V. (1996). Perenos Ionov v Membranakh.

[52] Dobrevsky I., Zvezdov A. (1979). Investigation of Pore Structure of Ion Exchange Membranes. Desalination.

[53] Belashova E.D., Minakova E.A., Kharchenko O.A., Pismenskaya N.D. (2016). Influence of structural changes on current-voltage characteristics of the anion exchange membrane after their long contact with an ampholyte solution. Sorptsionnye Khromatographicheskie Protsessi.

[54] Fieser L.F., Fieser M. (1961). Organic Chemistry: Advanced Course.

[55] Selemenev V.F., Rudakov O.B., Slavinskaya G.V., Drozdova N.V. (2008). Pigments of Food Production (Melanoidins).

[56] Ruban E.L., Liakh S.P., Khruleva I.M., Titova I.A. (1969). [Melanin pigments in Nadsoniella nigra]. Izv. Akad. Nauk. SSSR Biol..

